# Influence of Loading Density and Gender on the Welfare and Meat Quality of Horses During Transport for Slaughter

**DOI:** 10.3390/ani14213069

**Published:** 2024-10-24

**Authors:** Vesna Božić Jovanović, Ružica Trailović, Ivan Vićić, Nevena Grković, Milena Radaković, Nedjeljko Karabasil, Ana Kaić, Nikola Čobanović

**Affiliations:** 1 Department of Food Hygiene and Technology, Faculty of Veterinary Medicine, University of Belgrade, Bulevar Oslobodjenja 18, 11000 Belgrade, Serbia; bozicvesna@hotmail.com (V.B.J.); ivan.vicic@vet.bg.ac.rs (I.V.); nevena.ilic@vet.bg.ac.rs (N.G.); nedja@vet.bg.ac.rs (N.K.); 2 Department of Animal Breeding, Faculty of Veterinary Medicine, University of Belgrade, Bulevar Oslobodjenja 18, 11000 Belgrade, Serbia; ruzicat@vet.bg.ac.rs; 3 Department of Pathophysiology, Faculty of Veterinary Medicine, University of Belgrade, Bulevar Oslobođenja 18, 11000 Belgrade, Serbia; mradakovic@vet.bg.ac.rs; 4 Department of Animal Science and Technology, Faculty of Agriculture, University of Zagreb, Svetošimunska Cesta 25, 10000 Zagreb, Croatia; akaic@agr.hr

**Keywords:** acute-phase response, blood metabolites, carcass bruises, Domestic Mountain Pony, horsemeat quality, oxidative stress biomarker

## Abstract

The aim of this study was to determine the effects of loading density and gender on blood welfare indicators, carcass bruises and horsemeat quality. Data were collected from twelve transports of 89 slaughter horses originating from the same collection centre. The transportation of slaughter horses at high loading densities (>200 kg/m^2^), especially stallions, resulted in increased levels of lactate (*p* = 0.021), glucose (*p* < 0.0001), ceruloplasmin (*p* < 0.0001) and AOPP (*p* < 0.0001), but lower GSH levels (*p* < 0.0001). Compared to stallions and mares, geldings subjected to high loading density (>200 kg/m^2^) during transport had lower levels of the aforementioned blood metabolites. In addition, stallions exposed to a high load density (>200 kg/m^2^) had the highest frequency of severe (*p* = 0.0002), large (*p* < 0.0001) and circular (*p* = 0.0001) carcass bruises, which were predominantly located on the abdominal (*p* = 0.0056) and thoracic (*p* = 0.0004) wall. In contrast, a higher percentage (*p* < 0.0001) of undamaged carcasses was found in slaughter horses exposed to a low loading density (≤200 kg/m^2^) during transport. Stallions exposed to high loading densities (>200 kg/m^2^) during transport had the highest initial pH (*p* < 0.0001) and ultimate pH (*p* = 0.005) in terms of *m. longissimus lumborum* and redness (*p* = 0.017), but the lowest drip loss (*p* = 0.050) and lightness (*p* < 0.0001), which, consequently, led to the highest (*p* = 0.0045) proportion of DFD-like defects in the meat quality. In conclusion, the results of this study showed that high loading densities (>200 kg/m^2^), regardless of gender, negatively affect horse welfare during transport. In addition, stallions were more sensitive to poor pre-slaughter conditions and produced the lowest meat quality, while geldings were the most resistant. To determine the optimal transport density, further research is needed to determine the effects of different loading densities on the behaviour, physiology, carcass and meat quality of slaughter horses.

## Simple Summary

The transportation of horses for slaughter is a complex process and represents a significant health, welfare and ethical problem. During transport, horses for slaughter are exposed to various stress factors that could affect their welfare, health and performance, as well as the profitability of the industry. To date, there are no data on the response of slaughter horses exposed to different loading densities during transport. The aim of this study was therefore to determine the effects of loading density and gender on blood components, carcass bruises and the meat quality of slaughter horses. The results of this study showed that transport with a high loading density (>200 kg/m^2^), especially for stallions, had a negative effect on horse welfare. In contrast, transporting horses for slaughter at lower loading densities (≤200 kg/m^2^), regardless of gender, led to an improvement in their welfare. Meat from stallions transported at high loading densities (>200 kg/m^2^) had the highest pH, lower water-holding capacity, an unfavourable dark red colour and the highest percentage of DFD-like meat quality defects. In contrast, mares and geldings had higher meat quality in terms of pH, with a higher water-holding capacity and better colour, regardless of loading density. In conclusion, stallions were the most sensitive to poor transportation conditions and had the lowest meat quality.

## 1. Introduction

The transportation of horses, especially those destined for slaughter, poses a significant health and welfare problem [1,2]. Several risk factors for transport-related welfare problems have been identified, such as inadequate monitoring of fitness prior to transport, lack of or insufficient supply of water and feed, high loading densities, transport duration/distance, and elevated temperatures inside the lorry, suggesting that the transportation of horses for slaughter is a complex process [2,3]. To ensure the welfare of horses for slaughter and optimise meat quality, every stage of transport must be carried out with the greatest care, as excessive stress can lead to bruising, injuries and deterioration of horsemeat quality [3]. It has been reported that most horses transported for slaughter are usually collected by traders from various sources and/or purchased at auction markets in order to collect enough animals to fill a lorry for transport to a slaughterhouse [3,4]. In addition, horses of different genders are usually transported from farms/collection centres to the slaughterhouse at the end of their primary use and may, therefore, react differently to stressful situations on the day of slaughter [5,6]. Under commercial conditions, the proportion of stallions within a load of slaughter horses is usually low (no more than two) compared to mares (50 to 58%) and geldings (40 to 47%), because they are more aggressive and more difficult to handle, but also due to the low number of non-castrated horses in the total population [3,4]. Although stallions are usually more bad-tempered, dominant, aggressive and difficult to handle [3,4], geldings can also express agonistic behaviour, while mares can be aggressive towards other animals and humans after parturition as a natural behaviour to protect their foal [7]. In addition, many of these horses are reported to be untrained (unhandled) and transported in poor conditions, raising further ethical concerns [2].

In Europe, European Regulation 1/2005 [8] establishes the legal conditions for the transportation of animals, which must be complied with, depending on the species and category of animals being transported. In the case of horses, the regulation only permits the transportation of handled horses for more than eight hours, while unhandled horses are limited to a maximum transport time of eight hours. In addition, unhandled horses must be transported in small groups of no more than four animals per compartment, as they find it difficult to cope with a new environment, being tied or isolated [2]. Furthermore, horses must not be transported on the upper decks of multi-deck transport vehicles, and the height above the withers of the tallest individual must be at least 75 cm [1]. Various authorities and guidelines have defined specific ranges for the optimum loading density for horse transport [3]. For example, the European Regulation 1/2005 [8] mandates a minimum space allowance of 1.75 m^2^ for an adult horse during longer journeys, while the Australian Animal Welfare Standards and Guidelines [9] stipulate a minimum space allowance of 1.2 m^2^ for the same conditions in Australia. Nevertheless, the proposed and/or legislated loading densities for animal transport are sometimes not met in practice [4]. Although loading density is one of the most easily influenced variables during transport, its importance is usually overlooked and lorries are frequently overloaded; the number of slaughter animals is increased per load to maximise transport profit, creating an economic burden. To date, there is no consensus on specific recommendations for optimal loading densities in lorries during the transportation of horses for slaughter based on extreme weather conditions, live weight and transport time to ensure transport economy, animal health and welfare, and, in particular, high carcass and meat quality.

Compared to the transportation of sport and leisure horses [10,11,12], the transportation of horses for slaughter under commercial conditions and their responses to stressful situations have received less research attention [11,12,13,14,15]. Several studies have shown that transportation with too-high or too-low loading densities has a negative effect on the welfare of horses for slaughter [13,14]. Interestingly, the influence of loading density on meat quality in cattle [16,17], fattening pigs [18,19,20], lambs [21,22,23] and broiler chickens [24,25] has been extensively investigated. Several studies identified the differences between male and female horses in carcass and meat quality traits [6,26,27,28], while information on the effect of transport conditions on the different gender classes is scarce. As far as the authors are aware, the effects of loading density and gender on physiological stress indicators, carcass bruises and meat quality characteristics of slaughter horses have not yet been investigated in a single study. Considering the knowledge gaps described above, the aim of this study was to determine the effects of loading density and gender on blood welfare indicators, carcass bruises and the meat quality of slaughter horses.

## 2. Materials and Methods

### 2.1. Ethical Statement

The study was carried out in accordance with the Guide to Good Animal Welfare Practice for the Keeping, Care, Training and Use of Horses [29], European legislation on the protection of animals during transport (Council Regulation-ECN 1/2005 [8]) and at slaughter (Council Regulation-EC-N 1099/2009 [30]). The horses for slaughter originated from the same collection centre and were slaughtered for human consumption in the same accredited slaughterhouse (Pećinci, Srem district, Serbia). The horses were not subjected to any experimental invasive procedures in vivo (blood samples were taken during exsanguination, while bruises on the carcasses were recorded in the cooling room). In addition, the data collection was intentionally carried out under standard commercial pre-slaughter conditions. Consequently, no specific instructions were given to the lorry driver, the loading staff or the slaughterhouse personnel regarding the handling of horses for slaughter. Therefore, this study did not fall within the scope of Directive 2010/63/EU [31] on the protection of animals used for scientific purposes and was therefore exempt from specific authorisation by the local animal welfare and ethical review body.

### 2.2. Experimental Animals, Pre-Slaughter Conditions and Slaughter Procedure

A total of 89 slaughter horses (35 mares, 31 geldings and 23 stallions) with an average live weight of 339.50 ± 76.59 kg and a slaughter age of 3.05 ± 0.76 years (ranging from two to four years) were evaluated in twelve shipments during spring. All slaughter horses were of the same breed (Domestic Mountain Pony) and originated from the same collection centre (Ruma, Srem district, Serbia), where they were kept under uniform and standardised husbandry conditions. The collection centre is a small family horse farm where the animals are usually purchased from various sources and/or auction markets and fed concentrates and forage that meet the National Research Council’s nutritional recommendations for horses until slaughter [32]. At the collection centre, 30 to 50 domestic horses (Domestic Mountain Pony) were kept for a short period (approximately one month) in the two stables, which consisted of 25 separate stalls (3.0 × 3.0 m and natural ventilation) for individual animals. The floors of the stables were made of non-slip concrete, designed to allow the horse’s urine to drain away (with drainage channels) and covered with a thin layer of straw. Each stable had a run-out area of 1000 m^2^ (50 × 200 m), with the stable gates usually left open to allow free outdoor access. Clean, fresh water was constantly provided in automatic drinking bowls in each stall. Hay was available ad libitum in hay nets attached at the horses’ head height, while concentrates were given in two equal meals at 08:00 h and 17:00 h. The slaughter horses were not fasted prior to transport but had unrestricted access to drinking water throughout their stay at the collection centre. The horses were slaughtered in March, April and May (four shipments per month, once a week). A shipment was defined as “a group of horses that were exposed to the same conditions before slaughter (originating from the same collection centre, transported in the same lorry at the same time and exposed to the same lairage and slaughter conditions)”.

At the collection centre, the horses were loaded by the lorry driver (owner) one after the other with halter and lead rope over the rear ramp of a standard single deck lorry. Loading times varied between 15 and 32 min (average 23.00 ± 4.99), and the lorry departed immediately after the loading was completed. All journeys were conducted using the same lorry, with the same driver, driving style and route. The distance from the collection centre to the same commercial slaughterhouse was 60 km, with a transport time of approximately one hour (58.92 ± 5.468 min). The loading density varied between 165.40 and 254.73 kg/m^2^, depending on the live weight and number of slaughter horses in the transport vehicle. Feed and water were not available during transport. During the sampling days, the ambient temperature remained within the thermoneutral zone for horses, ranging from 16.0 °C to 21.0 °C [33], while the relative humidity fluctuated between 42.0% and 58.4%. The temperature–humidity index also stayed within the normal range for this species, between 59.94 and 66.54 [34]. Upon arrival at the slaughterhouse, the horses were immediately unloaded individually by the lorry driver, with a waiting time of between 10 and 25 min (average 17.58 ± 5.09). The individual consignments were unloaded using a lorry ramp at a 10° angle, with a non-littered floor. Unloading times varied between 20 and 38 min (average 27.17 ± 5.39). The horses of the same consignment were driven 10 m to the covered lairage pens (lairage density of 2 m^2^/individual), where they rested in the same groups for three hours (181.90 ± 6.59 min), assigned by the veterinarian in charge depending on the physical condition of the animals and the weekly schedule of the slaughterhouse. During the resting period, the horses were provided with easily accessible drinking water, but no access to feed was provided. The temperature and relative humidity in the lairage pens corresponded to the external conditions. Official veterinary inspectors carried out antemortem inspections in the lairage pens and found no clinical signs of disease in any of the examined horses. The horses were transported and kept in lairages without being separated by gender. After unloading the group of animals and moving them to the lairage pens, each selected horse was assigned a traceability number by the slaughterhouse, a traceability sheet was completed, and it was weighed. The assigned traceability number was used to identify and track the horses from the pre-slaughter stage to the cooling room.

The research team consistently followed the arrival of the horses at the slaughterhouse and conducted brief interviews with the lorry driver. The aim of these interviews was to collect information on the number of horses transported, the collection centre of origin, the daily temperature and relative humidity during loading, the loading time, the loading density, the journey time, the distance from the collection centre to the slaughterhouse, the waiting time and the unloading time. The research team then ensured that the slaughter horses declared by the lorry driver corresponded to those actually received. In addition, information was obtained from the slaughterhouse staff about lairage time, lairage density, daily temperature and relative humidity during unloading and lairage. These data were used to create a database for the identification of horse carcasses in the cooling room during the evaluation of bruises, carcass and meat quality assessment. The temperature–humidity index (THI) was calculated based on the ambient temperature (AT°; expressed in °C) and relative humidity (RH; expressed as a fraction of a unit) during loading, unloading and at lairage by using the following formula: THI = (1.8 × AT° + 32) − [0.55 − 0.55 × (RH/100)] × [1.8 × AT° − 26] [34]. A THI of 70 or lower indicates a non-stressful environment, while a THI of 71 to 78 is classified as critical, 79 to 83 is deemed dangerous, and values exceeding 83 indicate an emergency situation [34]. The summarised information on pre-slaughter conditions can be found in Table 1.

After lairage, the slaughter horses were stunned with a captive bolt pistol, exsanguinated by cutting the neck blood vessels (*a. carotis communis* and *v. jugularis*) and dressed in line with the applicable European Union regulations [30].

### 2.3. Collection, Selection, Preparation of Blood Samples and Determination of Selected Blood Components

After bleeding started, blood samples were taken from each horse. Blood levels of lactate and glucose were measured at the slaughter line using portable devices (blood glucose: Accu-chek^®^ Performa, Roche Diagnostics, Mannheim, Germany; blood lactate: Accutrend Plus, Roche Diagnostics, Roche, Mannheim, Germany). For the determination of acute-phase proteins and oxidative stress biomarkers, blood samples were kept refrigerated (3 ± 1 °C) until they were processed immediately upon arrival at the laboratory. The concentrations of ceruloplasmin and haptoglobin in plasma were determined according to the methods of Hussein et al. [35] and Jones et al. [36], respectively. Plasma levels of reduced glutathione (GSH), advanced oxidation protein products (AOPP), total antioxidant capacity (TAC) and total oxidative stress (TOS) were determined according to the methods of Ellman and Jollow et al. [37,38], Witko-Sarsat et al. [39] and Erel [40,41], respectively. The Oxidative Stress Index (OSI) was determined by calculating the combined ratio of TOS to TAC.

### 2.4. Evaluation of Bruises on the Carcass

**Figure 1:**
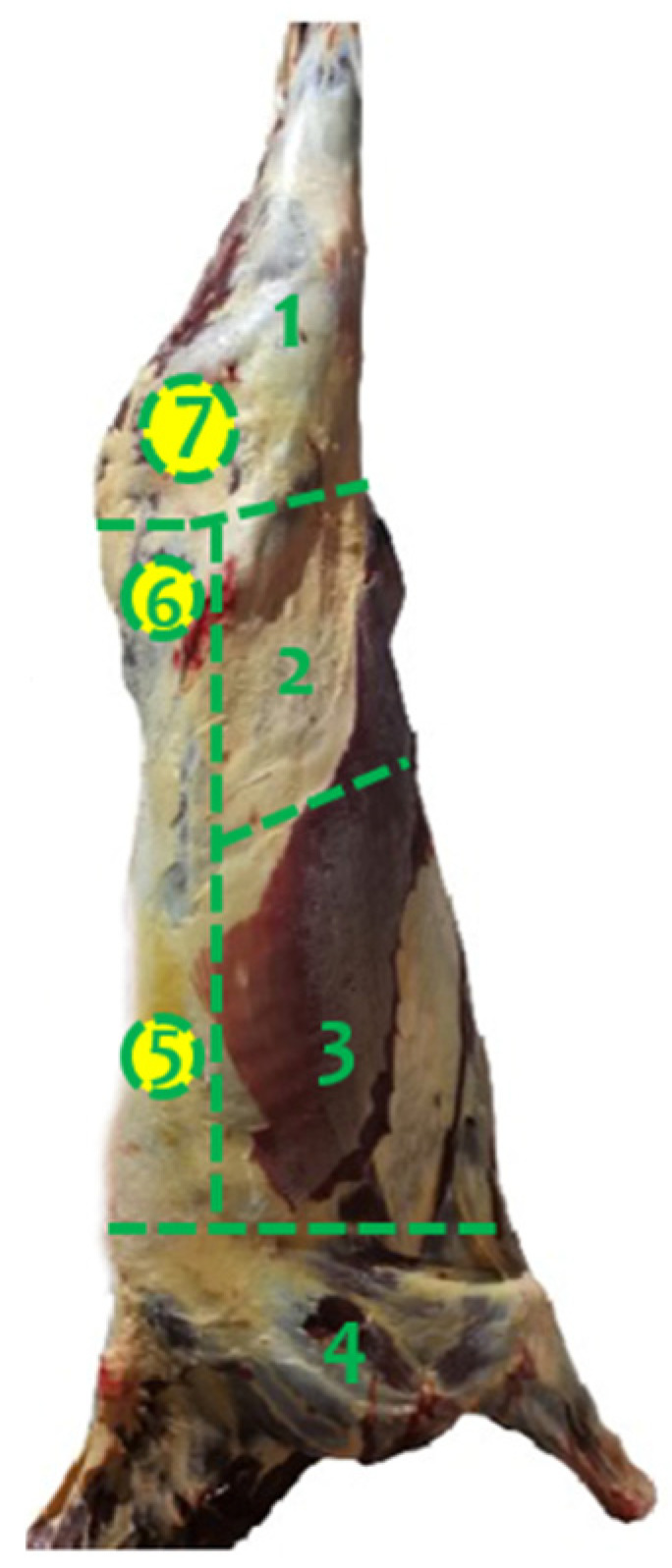
Different anatomical regions of the horse carcass used for the documentation of bruises as follows: 1= rear limb, 2 = abdominal wall, 3 = thoracic wall, 4 = front leg, 5 = loin, 6 = Tuber coxae and its muscular insertions, 7 = Tuber isquiadicum and its muscular insertions.

**Figure 2:**
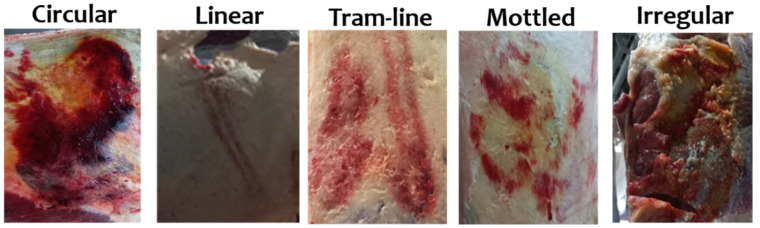
Classification of bruises on horse carcass based on the shape.

The bruises on the carcasses were assessed 45 min after slaughter in the cooling room. Both sides of the carcasses were assessed using a visual scoring system based on the methodology of Miranda-de la Lama et al. [42]. First, the presence of bruises on each carcass was documented as present or absent. When bruises were detected, the observer recorded both the total number of bruises per carcass and the number per specific anatomical site (Figure 1). In addition, each individual bruise on the carcass was scored, documenting details such as severity (grade 0 = no visible bruises; grade 1 = involves affecting subcutaneous tissue; grade 2 = involves subcutaneous tissue and muscle; grade 3 = involves subcutaneous tissue, muscle, and bone), shape (circular, linear, tramline, mottled, and irregular; Figure 2), and size (small = ≥5 cm in diameter; medium = 6–10 cm; and large = ≥10 cm).

### 2.5. Meat Quality Assessment

Meat quality was assessed on the left side of the carcass at three different time points as follows: 45 min, 24 h and 72 h post mortem. The same research team, consisting of two individuals, carried out all measurements of the meat quality parameters. The coefficients of variation within the measurements for all meat quality parameters analysed remained consistently below 10%.

The pH and temperature of the *m. longissimus lumborum* (between the 12th and 14th rib at a depth of 5 cm) and *m. gracilis* (5 cm above the bone and at a depth of 3 cm) were determined using a digital portable pH meter (Testo 205, Testo AG, Lenzkirch, Germany) after calibration with pH 4.0 and 7.0 buffer solutions. The initial pH (pH_i_) and temperature (T_i_) in each muscle were measured 45 min post mortem at the slaughter line, directly on the carcass before cooling. The ultimate pH (pH_u_) and temperature (T_u_) in each muscle were determined 24 h after slaughter directly on the carcass in the cooling room (24 h after chilling). Both the meat pH and temperature were measured three times for each sample, and the average of these three measurements was used as the final result.

Trained slaughterhouse personnel then cut three boneless loin samples (MLL) from each selected carcass 24 h post mortem, exactly at the level of the 13th and 14th rib. These samples were then deboned, weighed on an electronic scale (WPS 600/C, Radwag, Radom, Poland) and standardised into three 2.54 cm (100 ± 1.5 g) thick steaks. The meat samples intended for analysis of water-holding capacity and colour were then vacuum-packed, placed in a cool box on shaved ice (at 3 ± 1 °C) and transported to the laboratory for further analysis. Objective meat colour was determined using a portable colorimeter (NR110, 3NH Technology Co., Ltd., Shenzhen, China), with a 4 mm aperture, a viewing angle of 2° and a D65 illuminant. Measurements were taken after 30 min of blooming at room temperature (21–23 °C) at nine randomly selected points on the surface of the loin muscle and in the core after slicing to obtain a representative average value for colour [43]. The final colour result was determined by averaging the values of L* (lightness), a* (redness) and b* (yellowness) from the nine measurements. The water-holding capacity of the meat samples was evaluated using drip loss, thawing loss and cooking loss. Each method was performed in triplicate, following the guidelines provided by Klauke et al. [44]. According to Čobanović et al. [45], the quality classes of horsemeat were categorised based on the pH value measured 24 h after slaughter in the MLL, as follows: (i) acid meat, with pH_u_ values below 5.4; (ii) normal-quality meat, with pH_u_ values ranging from 5.4 to 5.9; and (iii) dark, firm and dry (DFD)-like meat, with pH_u_ values exceeding 6.0.

### 2.6. Statistical Analysis

The statistical analysis of the results was carried out using SPSS software, version 23.00 for Windows [46]. Before conducting the statistical analysis, tests were carried out to assess linearity, normality of residuals (Shapiro–Wilk and Kolmogorov–Smirnov test), outliers, and homogeneity of variance (Levene’s test) for the dependent variables. The data met all the specified criteria for these tests. The slaughter horses were divided into three groups according to gender, as follows: mares = defined as female, fully developed and ready for breeding (*n* = 35), geldings = defined as castrated male (*n* = 31), and stallions = defined as non-castrated, i.e., intact, ready for breeding male (*n* = 23). According to the loading density, the slaughter horses were divided into two categories based on the threshold value proposed by Weeks et al. [1] for an appropriate maximum loading density (200 kg/m^2^) for ponies transported in groups, as follows: high loading density = space available in the transport vehicle above 200 kg/m^2^ (*n* = 50); low loading density = space available in the transport vehicle bellow 200 kg/m^2^ (*n* = 39).

A General Linear Mixed Model analysis was used to analyse the influence of transport time, loading density, lairage time, gender and age (2 × 2 × 2 × 2 × 2) as fixed effects on the selected blood constituents and meat quality traits of slaughter horses. All two-, three-, four-, and five-way interactions between the fixed effects were initially analysed and excluded from the model if *p* > 0.05. The two-way interaction (loading time × gender) was significant and was therefore retained in the final model to elucidate significant effects. Comparisons between groups for all parameters analysed (with the exception of carcass bruise characteristics and meat quality classes) were assessed using one-way analysis of variance (ANOVA), followed by Tukey’s multiple comparison tests, with differences considered significant at *p* < 0.05. Data were presented as means with standard deviations. Significant differences between groups in terms of carcass bruises and meat quality classes between groups were analysed using the Chi-squared test. Statistical significance was recognised at *p* < 0.05, with trends considered at 0.05 < *p* < 0.10.

## 3. Results

### 3.1. The Effects of Loading Density and Gender on Selected Blood Components of Slaughter Horses

**Figure 3:**
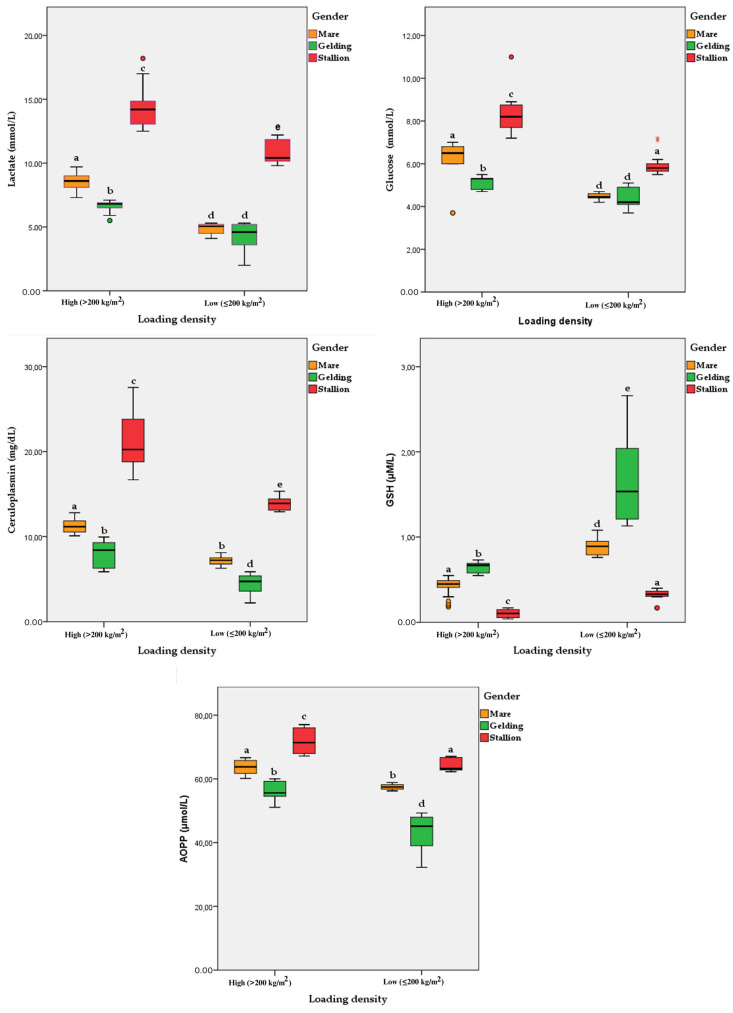
Boxplots of the interactive effects of loading density and gender on selected blood components of slaughter horses. The whiskers represent the ranges for the bottom 25% and the top 25% of the data values, while bold horizontal lines represent the median values. Circles and stars outside the whisker represent outliers (95%). Note: *p*-values correspond to two-way ANOVA followed by Tukey’s multiple comparison test; different letters indicate significant differences at *p* < 0.05 ^(a–e)^.

The effects of loading density and gender on selected blood components of slaughter horses are shown in Table 2. The values of lactate, glucose, ceruloplasmin, GSH and AOPP differed (*p* < 0.0001) according to loading density during transport. Gender affected (*p* < 0.0001) the concentrations of lactate, glucose, ceruloplasmin, GSH and AOPP. The interaction between loading density and gender affected the levels of blood metabolites, plasma acute-phase proteins and oxidative stress biomarkers. The highest concentrations of lactate (*p* = 0.021), glucose (*p* < 0.0001), ceruloplasmin (*p* < 0.0001), GSH (*p* < 0.0001) and AOPP (*p* < 0.0001) were recorded in stallions exposed to high loading densities (>200 kg/m^2^) (Figure 3).

### 3.2. The Effects of Loading Density and Gender on the Occurrence of Carcass Bruises of Slaughter Horses

The effects of loading density and gender on the occurrence of carcass bruises in slaughter horses are shown in Table 3. Most carcass bruise characteristics differed according to loading density during transport, with the exception (*p* > 0.05) of bruise size, while gender affected all carcass bruise characteristics. The interactive effect of loading density and gender affected the occurrence of carcass bruises in slaughter horses; the highest percentages of severe (*p* = 0.0002), large (*p* < 0.0001) and circular (*p* = 0.0001) carcass bruises, predominantly located on the abdominal (*p* = 0.0056) and thoracic (*p* = 0.0004) wall, were found in stallions subjected to a high loading density (>200 kg/m^2^).

### 3.3. The Effects of Loading Density and Gender on the Horsemeat Quality

**Figure 4:**
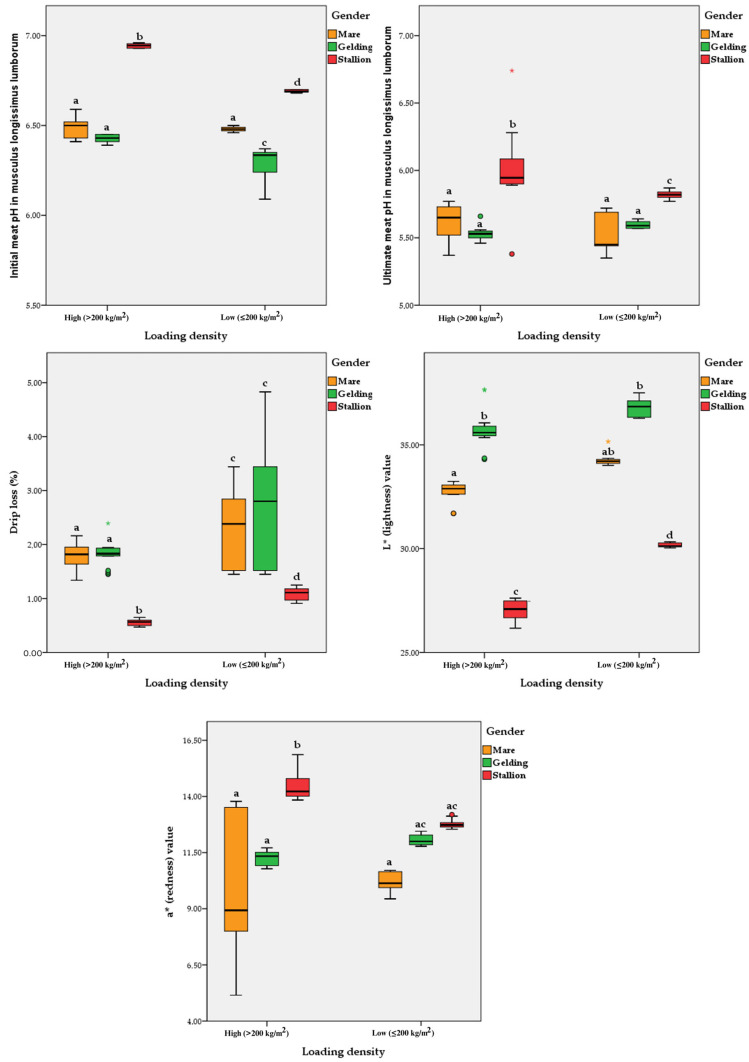
Boxplots of the interactive effects of loading density and gender on horsemeat quality. The whiskers represent the ranges for the bottom 25% and the top 25% of the data values, while bold horizontal lines represent the median values. Circles and stars outside the whisker represent outliers (95%). Note: *p*-values correspond to two-way ANOVA followed by Tukey’s multiple comparison test; different letters indicate significant differences at *p* < 0.05 ^(a–d)^.

The effects of loading density and gender on the horsemeat quality are shown in Table 4. The initial (pH_i_) (*p* < 0.0001) and ultimate (pH_u_) (*p* = 0.021) meat pH in *m. longissimus lumborum*, initial meat temperature (T_i_) in *m. gracilis* (*p* = 0.004), drip loss (*p* < 0.0001), thawing loss (*p* = 0.017), L* (lightness) (*p* < 0.0001) and b* (yellowness) (*p* = 0.004) values in *m. longissimus lumborum* significantly differed with loading density during transport. Gender had a significant effect on several meat quality parameters, including the initial and ultimate meat pH (*p* < 0.0001) in *m. longissimus lumborum*, the initial meat temperature (*p* = 0.050) in *m. gracilis*, drip loss (*p* < 0.0001), as well as the L* (lightness), a* (redness), and b* (yellowness) values (*p* < 0.0001) in *m. longissimus lumborum*, and the incidence of DFD-like meat (*p* = 0.0035). The interaction between loading density and gender significantly influenced most horsemeat quality characteristics. Stallions subjected to high loading densities (>200 kg/m^2^) had the highest initial (*p* < 0.0001) and ultimate (*p* = 0.005) meat pH, as well as a* higher (redness) value (*p* = 0.017) in *m. longissimus lumborum* (Figure 4). Conversely, they showed the lowest drip loss (*p* = 0.050) and L* (lightness) value (*p* < 0.0001) in *m. longissimus lumborum*. In addition, the highest percentage of DFD-like meat (*p* = 0.0045) was found in stallions with a high load density (>200 kg/m^2^) (Figure 4).

## 4. Discussion

### 4.1. The Effects of Loading Density and Gender on Welfare of Slaughter Horses

Each stage of transport subjects horses to a number of stressful factors, which can be psychological (separation or mixing of unfamiliar individuals, inappropriate loading density, agonistic behaviour, human–animal interaction, and exposure to novel surroundings) or physical (prolonged water and feed deprivation, noise, vibration, acceleration, exhaustion, carcass bruises, and adverse weather conditions) [47]. Therefore, any period of transport, especially under inadequate conditions, can induce different degrees of stress in horses, ranging from mild discomfort to extreme distress (and even death), leading to activation of oxidative stress and acute-phase response and changes in the concentration of blood metabolites, enzymes and hormones [4,15,47]. Earlier investigations [48,49,50] have reported that oxidative stress biomarkers such as GSH, AOPP, TAC, TOS and OSI) and acute-phase proteins (including haptoglobin and ceruloplasimin) have a great potential to be reliable and accurate indicators for monitoring the stress level experienced by the horses. Nevertheless, none of the currently available studies examined the effect of loading density and gender on the physiological stress indicators of horses during transport for slaughter under commercial conditions.

Analysis of blood constituents revealed that transport at high loading densities (>200 kg/m^2^), especially stallions, resulted in increased lactate, glucose, ceruloplasmin and AOPP levels, but decreased GSH levels (Table 1), suggesting activation of the acute-phase response and induction of oxidative stress, thus seriously compromising welfare during transport. Compared to stallions and mares, geldings exposed to high loading densities (>200 kg/m^2^) during transport had lower concentrations of the aforementioned blood metabolites, acute-phase proteins and oxidative stress biomarkers, with the exception of GSH, whose concentration was higher (Table 1), suggesting a milder stress response and higher resistance to poor transport conditions. In contrast, transport at low loading densities (≤200 kg/m^2^), especially in geldings, resulted in lower lactate, glucose, ceruloplasmin and AOPP levels, but higher GSH levels (Table 1), indicating a lower acute-phase response and lower oxidative stress, and thus better welfare conditions during transport.

A much stronger stress reaction of slaughter horses transported at high loading density could be explained by the fact that horses need more space during the journey than just the contours of their body, as they have a high centre of gravity and 60% of their weight is supported by the forelegs [3,51]. To effectively manage acceleration and deceleration during movement, horses extend their feet beyond the typical position under their body, creating a wider base [3]. They also adjust their head and neck posture in the direction they are facing to maintain balance [3,52,53]. In addition, horses endeavour to avoid contact with other individuals and the sides of the transport vehicle during transport [11,54,55,56]. Therefore, it can be argued that these adjustments, ensuring stability and reducing stress, require horses to have more floor space in the lorry during transport than when standing still. Some authors [14] also reported that the horses in the higher stocking density groups may have been subjected to increased stress due to their inability to escape, possibly leading to a state of learnt helplessness, which further explains the results of the present study.

Considering that stallions are generally larger, more dominant and more temperamental than mares and geldings [5,6], a much stronger stress response during transport in a crowded transport vehicle could be expected, which is also confirmed in the present study (Table 2). Previous studies [5,6] reported that stallions are more sensitive to stress. Another reason for a higher stress response in horses transported at high loading densities could be the mixing of unfamiliar horses during the journey. Social animals by nature, temperamental and dominant horses, like stallions, can become nervous and agitated during transport, potentially affecting the behaviour of neighbouring horses in the lorry [3]. In addition, previous reports [57,58] have shown that anxiety and arousal can be socially transmitted among herd members, which should be taken into account when mixing horses with different temperaments and travelling experiences [59,60]. In contrast, the greater resilience of geldings to adverse transport conditions may be attributed to the effects of castration, which likely alters their behaviour due to the absence of androgens. This results in geldings spending more time standing, showing less interest in exploring new environments, forming stronger bonds with handlers, and responding more calmly to unfamiliar stimuli on the day of slaughter [7].

Carcass bruises serve as important forensic indicators for identifying pre-slaughter logistic chain failures, such as electric goad use, slipping/falling, rough handling, mixing of unacquainted animals, and/or facility infrastructure and/or transport vehicle hazards like rough edges and drop gates [42]. Furthermore, the presence of bruises on horse carcasses is of significant concern for both the meat industry and the public, as it is linked to animal abuse and suffering, excessive carcass trimming, deterioration in meat quality and freshness, and potential financial losses [42]. In the present study, slaughter horses of all genders exposed to a high loading density (>200 kg/m^2^) had a higher frequency of mild, circular carcass bruises, typically located on the abdominal and thoracic wall (Table 2). In addition, stallions exposed to the same transport conditions had the highest frequency of severe, large and circular carcass bruises, which were predominantly located on the abdominal and thoracic wall (Table 2). In contrast, a higher percentage of undamaged carcasses was found in slaughter horses exposed to a low loading density (≤200 kg/m^2^) during transport (Table 2).

It has been reported that high loading densities increase the risk of horses slipping and falling during transport, which can lead to injuries from biting, kicking, toppling and trampling [3,13]. Similarly, invasion of another horse’s personal space is a common trigger for aggression, so providing less space per individual during transport can be expected to increase the level of aggression in horses [61]. However, it is important to emphasise that aggressive behaviour during transport is related to the temperament of individual horses rather than the specific conditions of transport [14,47]. This could also explain the highest incidence of carcass bruises in stallions, which is usually due to fighting, kicking or other physical interactions during loading, transport, unloading and/or at the lairage [42]. Stallions are typically more aggressive and challenging to handle than mares and geldings [3,4,5,6], a behaviour attributed to the effects of testosterone, which has been shown to stimulate agonistic behaviour through its influence on the brain [7,62]. Another reason for a higher frequency of injuries during transport at high loading density could be the mixing of unfamiliar horses of different genders and weights; it is not uncommon under commercial conditions to form batches adapted to the size of lorry [3]. Conflicts between un(familiar) animals of the same or different species are inevitable when exposed to a hostile or unfamiliar environment [63]. Regrouping horses prior to loading disrupts social cohesion among conspecifics in their original groups [3,64], which can lead to fighting (such as pushing, biting, kicking, bucking and head-tossing) as they establish a new social hierarchy, and causes bruising to their bodies, which is more pronounced in loosely transported groups [3,65]. During transportation, aggression in mixed groups can lead to sudden head movements and balance problems, increasing the risk of the horses’ heads striking the inside of the vehicle [3,4], which in turn contributes to the occurrence of bruises on the carcass. Aggressive social interactions during regrouping of horses also arise from competition for limited resources, such as water, feed and space [66]. As a stable social hierarchy is likely to help minimise aggressive behaviour among horses, frequent regrouping of unfamiliar individuals at the farm or collection centre should be avoided [3,63]. It has been suggested that horses need about seven days to become accustomed to new group members, as evidenced by a decrease in greeting behaviour and a smaller spacing between animals, suggesting that the group can be considered socially stable [66].

On the other hand, the lower frequency of carcass bruises in geldings exposed to high loading densities (>200 kg/m^2^) during the journey could be explained by the effect of surgical castration. It has previously been reported that horse aggression can be effectively controlled by castration [67,68]. In general, castration is expected to affect horse behaviour by eliminating the testicular production of androgens such as testosterone, which in turn affects other endocrine glands [68]. Testicular removal is expected to primarily affect aggression and reproductive behaviour [68]. Although castration has been shown to reduce aggressive behaviour in horses, geldings can still exhibit aggressive behaviour, but it is less frequent and typically less intense than that of stallions [7]. On the other hand, the low level of aggression and physical interactions during the transportation of slaughter horses at low loading densities can be attributed to the greater availability of floor space, which allows the animals to avoid each other [47,68]. Therefore, slaughter horses in less-crowded transport vehicles can cope better with temperament and dominant animals as they have the advantage of avoiding situations where biting and aggression occur, thereby reducing psychological and physical stress and injuries [13,14]. In this case, agonistic interactions between stallions and other dominant and temperamental individuals are regulated by ritualised displays and threat signals [68]. This is also confirmed in the present study, in which slaughter horses, regardless of gender, exposed to a low loading density (≤200 kg/m^2^) during transport had a higher percentage of undamaged carcasses than those exposed to a high loading density (>200 kg/m^2^) (Table 2). To improve the welfare of horses during the journey, geldings and mares should be transported at a lower loading density, while stallions and other temperamental and dominant horses should be isolated and kept in separate compartments within the lorry [3,8,69], which significantly improves transport management and reduces the risk of injuries from kicking and pawing [1,14]. The main reasons for transporting stallions to the slaughterhouse in a separate compartment from (sexually mature) mares and geldings are to prevent uncontrolled mating (especially during the breeding season) and the natural tendency of stallions to intermale aggression, as dominance hierarchies among male horses are more often contested compared to females [1,66]. Although only a few aggressive horses can cause most injuries during transport [70], it can be difficult to identify potentially aggressive animals before they are brought to the slaughterhouse; therefore, it is strongly recommended to load animals of a similar size, age and gender together in the lorry [1,3].

### 4.2. The Effects of Loading Density and Gender on the Horsemeat Quality Characteristics

Horsemeat is characterised by its dark red colour, yellow fat and high carbohydrate content (from intramuscular glycogen), along with a distinct sweet smell and taste [3,45]. The key factors influencing horsemeat quality include age, live weight at slaughter, breed, gender, production system, the sample anatomical location and pre-slaughter treatment [3,45]. There are three main criteria used in measuring the quality of horsemeat as follows: ultimate pH, water-holding capacity and instrumental colour [45]. Physicochemical indicators, such as meat pH and temperature, are crucial quality traits for horsemeat preservation and processing, as they help monitor the processes occurring during the postmortem transformation of muscle into meat [45]. Colour plays a vital role in the perception of meat quality and influences consumer purchasing decisions, as it serves as an indicator of the product’s freshness [45,71]. One of the most important properties influencing the economic value and quality of meat is its water-holding capacity, which determines the quality of fresh meat, meat yield, visual appeal, weight loss, cooking yield and sensory traits during consumption [45,72]. Previous studies have shown the negative influence of insufficient loading density during transportation on the quality characteristics of beef [16,17], pork [18,19,20], lamb [21,22,23] and broiler meat [24,25]. However, to the best of the author’s knowledge, this is the first study to analyse the effects of different loading densities and genders on the quality characteristics of horsemeat. The analysis of horsemeat quality revealed that slaughter horses exposed to high loading densities (>200 kg/m^2^) during transport had lower meat quality in terms of increased pH, a darker red colour and increased water-holding capacity, regardless of gender (Table 4). In addition, stallions exposed to high loading densities (>200 kg/m^2^) during transport had the slowest post mortem acidification (highest pH_i_ and pH_u_ in *m. longissimus lumborum*), an abnormally high water-holding capacity (lowest drip and thawing loss), the darkest red colour (lowest lightness and yellowness, but highest redness) and, consequently, the highest proportion of DFD-like meat quality defects (Table 4).

During transport, horses for slaughter are simultaneously exposed to a range of stressful situations, such as maintaining balance by constantly adjusting posture in response to vehicle movements during braking and/or cornering, being housed in unfamiliar spaces, mixing and fighting for space with unfamiliar conspecifics of different gender and weight, interacting with handlers, etc. [1,4,73]. These lead to excessive correction of muscle movements and require continuous energy expenditure, resulting in muscle fatigue and depletion of glycogen stores in skeletal muscles, limiting post mortem glycolysis and lactic acid production [74]. As a result, the pH of the meat does not decrease sufficiently, leading to a progressively higher pH, an unfavourable dark red colour and an abnormally increased water-holding capacity, which contributes overall to the occurrence of DFD meat [74]. Apart from the fact that DFD meat has many technological defects, this meat quality deficiency shows increased autolytic processes, increased susceptibility to bacterial growth and spoils relatively quickly under both aerobic and anaerobic conditions; therefore, it is not suitable for prolonged storage compared to normal-quality meat [74].

Compared to the stallions exposed to high load densities (>200 kg/m^2^), the stallions exposed to low loading densities (≤200 kg/m^2^) during transportation produced meat with a lower initial (6.94 ± 0.22 vs. 6.69 ± 0.10) and a lower ultimate pH (6.00 ± 0.31 vs. 5.81 ± 0.03), a lighter red colour (lightness: 27.04 ± 0.52 vs. 30.17 ± 0.11; redness: 14.44 ± 0.59 vs. 12.78 ± 0.21 and yellowness: 3.87 ± 1.41 vs. 5.42 ± 0.75) and higher drip (0.56 ± 0.06% vs. 1.09 ± 0.02%) and thawing (3.95 ± 1.50% vs. 6.80 ± 1.22%) loss, which resulted in a significantly lower occurrence of DFD-like meat (33.33% vs. 9.09%) (Table 4). This suggests that more space in the lorry during transport of stallions resulted in a significantly lower stress response and thus better meat quality. Although the meat from stallions exposed to low lading densities had better-quality characteristics (pH_u_ within the normal range for horsemeat and better water-holding capacity), the colour traits of the meat were still outside the range reported for male horses of similar age [75,76]. In addition, the meat of the aforementioned group of stallions had a darker red colour than that of mares and geldings, regardless of loading density during the journey (Table 4). The dark red colour of horsemeat, accompanied by a slight brown tinge resulting from the high concentration of the muscle pigment myoglobin (7.4 mg/g), is one of the most unfavourable characteristics for consumer acceptance [76,77]. In addition, gender has a significant effect on meat colour intensity, with entire males generally having darker meat than females and castrated males, which is likely due to higher myoglobin content due to greater physical activity [78,79]. These increased myoglobin levels in intact males may also contribute to lower colour stability of the meat [78], all of which combine to cause consumer rejection and negatively influence purchasing decisions. Based on the results of this study, it can be argued that the meat from stallions transported at both high (>200 kg/m^2^) and low (≤200 kg/m^2^) loading densities is of lower quality and may not meet the rigorous standards required for placement in the market or the production of premium products.

On the other hand, the meat of mares and geldings exposed to both high (>200 kg/m^2^) and low (≤200 kg/m^2^) loading densities showed better-quality characteristics compared to that of stallions (Table 4). The ultimate pH of meat from mares and geldings was in the normal range for horsemeat (5.4–5.9; [76]), while the colour and water-holding capacity characteristics were in the range reported for slaughter horses [75,76]. Accordingly, the meat of mares and geldings, regardless of the loading density, has adequate-quality characteristics and fulfils the quality standards for the marketing and production of high-quality products.

## 5. Conclusions

This study revealed that high loading densities (>200 kg/m^2^) during transportation significantly compromise the welfare of slaughter horses, particularly stallions, who exhibit heightened stress responses and a greater incidence of carcass bruises. In contrast, geldings display better resilience under these conditions. Additionally, the quality of horsemeat suffers, with high loading densities resulting in undesirable characteristics such as elevated pH levels and a darker meat colour. Future research should focus on determining optimal loading densities that ensure both animal welfare and meat quality, exploring strategies for reducing stress during transportation, and assessing the long-term effects of transport conditions on horse health and behaviour. Establishing clear guidelines will be essential for improving practices in the equine meat industry.

## Figures and Tables

**Table 1:** Pre-slaughter conditions of the twelve shipments of slaughter horses included in the study (*n* = 89).

Shipment	1	2	3	4	5	6	7	8	9	10	11	12
General information												
Number of horses	7	9	8	6	8	6	7	7	9	8	8	6
Average live weight per horse	315.17	353.79	329.49	363.13	344.48	358.45	338.00	345.50	296.10	359.79	338.38	344.58
Collection centre	Ruma	Ruma	Ruma	Ruma	Ruma	Ruma	Ruma	Ruma	Ruma	Ruma	Ruma	Ruma
Season	Spring	Spring	Spring	Spring	Spring	Spring	Spring	Spring	Spring	Spring	Spring	Spring
Month	March	April	April	March	May	April	March	March	May	May	May	April
Loading conditions												
Loading time (minutes)	19	29	25	18	23	20	22	28	32	20	25	15
Loading density (kg/m^2^)	183.85	254.73	210.88	174.30	220.45	179.22	189.28	193.48	213.16	234.86	216.56	165.40
Ambient temperature at loading (°C)	16.0	16.5	17.0	17.5	19.5	18.9	18.0	20.0	21.0	19.9	20.6	20.0
Ambient relative humidity at loading (%)	44.0	55.8	45.0	51.0	52.0	47.4	50.5	52.8	43.4	47.9	58.4	53.3
Temperature–humidity index at loading	59.94	60.80	61.21	62.02	64.70	63.70	62.66	65.40	66.13	62.00	66.54	65.43
Transport conditions												
Transport time (minutes)	62	60	63	58	55	68	50	54	59	66	60	52
Transport distance (km)	60	60	60	60	60	60	60	60	60	60	60	60
Unloading conditions												
Waiting time (minutes)	11	10	15	20	18	25	22	15	13	21	25	16
Unloading time (minutes)	24	35	30	22	28	20	25	30	38	25	27	22
Ambient temperature at unloading (°C)	16.5	16.9	17.5	18.8	20.5	20.0	19.1	20.5	20.5	20.9	20.0	20.2
Ambient relative humidity at unloading (%)	44.4	42.0	50.8	50.0	48.4	44.9	48.5	50.1	46.9	42.9	50.4	50.3
Temperature–humidity index at unloading	60.57	61.01	62.01	63.68	65.80	64.97	64.01	65.91	65.72	65.97	65.27	65.53
Lairage conditions												
Lairage time (minutes)	188	180	175	185	190	172	181	192	177	180	188	175
Lairage density (m^2^/horse)	2	2	2	2	2	2	2	2	2	2	2	2
Lairage temperature (°C)	16.8	16.2	16.9	17.8	19.1	19.5	18.5	19.5	19.0	20.0	19.2	18.9
Lairage relative humidity (%)	50.0	49.2	55.8	54.9	55.3	48.0	54.5	55.1	50.1	49.9	55.1	53.9
Temperature–humidity index at lairage	61.07	60.28	61.35	62.54	64.32	64.50	63.47	64.85	63.95	65.26	64.45	63.98

**Table 2:** Effect of loading density and gender on selected blood welfare indicators (mean value ± standard deviation) in slaughter horses (*n* = 89).

Loading Density	High			Low			Main Effects		Interaction
Gender	Mares	Geldings	Stallions	Mares	Geldings	Stallions	Loading Density	Gender	Loading Density × Gender
Number of Horses	21	17	12	14	14	11	*p*-Value		
Stress metabolites									
Lactate (mmol/L)	8.54 ± 0.71 ^a^	6.61 ± 0.50 ^b^	14.44 ± 1.72 ^c^	4.88 ± 0.43 ^d^	4.23 ± 1.19 ^d^	10.94 ± 0.97 ^e^	<0.0001	<0.0001	0.021
Glucose (mmol/L)	6.24 ± 0.92 ^a^	5.14 ± 0.27 ^b^	8.33 ± 1.02 ^c^	4.46 ± 0.14 ^d^	4.40 ± 0.47 ^d^	6.00 ± 0.60 ^a^	<0.0001	<0.0001	<0.0001
Acute-phase proteins									
Haptoglobin (g/L)	2.39 ± 0.23	2.41 ± 0.18	2.33 ± 0.39	2.37 ± 0.14	2.32 ± 0.27	2.41 ± 0.08	0.864	0.952	0.462
Ceruloplasmin (mg/dL)	11.26 ± 0.84 ^a^	7.99 ± 1.49 ^b^	21.21 ± 3.44 ^c^	7.24 ± 0.49 ^b^	4.45 ± 1.20 ^d^	13.93 ± 0.88 ^e^	<0.0001	<0.0001	<0.0001
Oxidative stress biomarkers									
GSH (µM/L)	0.42 ± 0.11 ^a^	0.65 ± 0.06 ^b^	0.10 ± 0.05 ^c^	0.89 ± 0.11 ^d^	1.64 ± 0.49 ^e^	0.31 ± 0.08 ^a^	<0.0001	<0.0001	<0.0001
AOPP (µmol/L)	63.35 ± 2.25 ^a^	56.10 ± 2.97 ^b^	71.90 ± 3.83 ^c^	57.49 ± 0.88 ^b^	43.13 ± 5.20 ^d^	64.33 ± 2.06 ^a^	<0.0001	<0.0001	<0.0001
TAC (mmol/L)	0.83 ± 0.34	0.70 ± 0.41	0.83 ± 0.40	0.73 ± 0.51	0.77 ± 0.53	0.93 ± 0.54	0.821	0.507	0.655
TOS (µmol/L)	84.16 ± 22.29	76.42 ± 22.10	78.87 ± 25.34	75.59 ± 26.39	71.06 ± 26.68	75.77 ± 24.57	0.285	0.601	0.914
Oxidative stress index	0.12 ± 0.06	0.16 ± 0.14	0.16 ± 0.18	0.31 ± 0.41	0.19 ± 0.21	0.16 ± 0.19	0.112	0.656	0.222

Abbreviations: high loading density—space available in the transport vehicle over 200 kg/m^2^ (*n* = 50); low loading density—space available in the transport vehicle equal to or less than 200 kg/m^2^; GSH—glutathione; AOPP—advanced oxidation protein products; TAC—total antioxidant capacity; TOS—total oxidative stress. Note: *p*-values correspond to two-way ANOVA followed by Tukey’s multiple comparison test; different letters in the same row indicate a significant difference at *p* < 0.05 ^(a–e)^.

**Table 3:** Effect of loading density and gender on the occurrence of carcass bruises in slaughter horses (*n* = 89).

Loading Density	High			Low				Main Effects		Interaction
Gender	Mares	Geldings	Stallions	Mares	Geldings	Stallions	Chi-square, df	Loading Density	Gender	Loading Density × Gender
Number of Horses	21	17	12	14	14	11		*p*-Value		
Bruise severity (%)										
No carcass bruises (grade 0) *	47.62 ^a^	58.82 ^a^	25.00 ^b^	85.72 ^c^	92.86 ^c^	81.82 ^c^	20.02, 5	<0.0001	0.2447	0.0012
Mild carcass bruises (grade 1)	42.86 ^a^	35.29 ^a^	16.67 ^ab^	7.14 ^b^	7.14 ^b^	9.09 ^b^	11.75, 5	0.0042	0.3827	0.0383
Moderate carcass bruises (grade 2)	9.52 ^a^	5.89 ^a^	58.33 ^b^	7.14 ^a^	0.00 ^a^	9.09 ^a^	24.66, 5	0.0599	0.0020	0.0002
Bruise size (%)										
Small (˂5 cm)	28.57	23.53	0.00	7.14	7.14	9.09	7.940, 5	0.1353	0.2448	0.1596
Medium (6–10 cm)	23.81	17.65	16.67	7.14	0.00	9.09	5.123, 5	0.0599	0.6735	0.4011
Large (≥10 cm)	0.00 ^a^	0.00 ^a^	41.67 ^b^	0.00 ^a^	0.00 ^a^	0.00 ^a^	33.99, 5	0.0649	0.0005	<0.0001
Bruise shape (%)										
Circular	14.29 ^a^	17.65 ^a^	58.33 ^b^	0.00 ^a^	0.00 ^a^	0.00 ^a^	25.19, 5	0.0004	0.0440	0.0001
Linear	9.52	5.88	0.00	7.14	7.14	9.09	1.250, 5	>0.9999	0.8187	0.9400
Tramline	9.52	5.88	0.00	7.14	0.00	9.09	2.466, 5	>0.9999	0.6125	0.7817
Mottled	9.52	5.88	0.00	0.00	0.00	0.00	4.549, 5	0.2531	0.4980	0.4733
Irregular	9.52	5.88	16.67	0.00	0.00	0.00	5.691, 5	0.0649	0.6887	0.3375
Anatomical region (%)										
Rear limb	4.76	5.88	0.00	0.00	0.00	0.00	2.800, 5	0.5020	0.6966	0.7308
Abdominal wall	4.76 ^a^	5.88 ^a^	33.33 ^b^	0.00 ^a^	0.00 ^a^	0.00 ^a^	16.47, 5	0.0330	0.0609	0.0056
Thoracic wall	4.76 ^a^	5.88 ^a^	41.67 ^b^	0.00 ^a^	0.00 ^a^	0.00 ^a^	22.62, 5	0.0167	0.0162	0.0004
Front leg	4.76	5.88	0.00	0.00	0.00	0.00	2.800, 5	0.5020	0.6966	0.7308
Loin	9.52	5.88	0.00	7.14	0.00	0.00	3.284, 5	0.6282	0.2789	0.6563
Hip	9.52	5.88	0.00	7.14	0.00	9.09	2.466, 5	>0.9999	0.6125	0.7817
Pin	9.52	5.88	0.00	0.00	7.14	9.09	2.466, 5	>0.9999	0.9459	0.7817

Abbreviations: *—expressed in relation to the total number of horses per group; high loading density—space available in the transport vehicle higher over 200 kg/m^2^ (*n* = 50); low loading density—space available in the transport vehicle equal to or less than 200 kg/m^2^; df—degrees of freedom. Note: *p*-values correspond to the Chi-squared test; different letters in the same row indicate a significant difference at *p* < 0.05 ^(a–c)^.

**Table 4:** Effect of loading density and gender on the horsemeat quality characteristics (*n* = 89).

Loading Density	High			Low			Main Effects		Interaction
Gender	Mares	Geldings	Stallions	Mares	Geldings	Stallions	Loading Density	Gender	Loading Density × Gender
Number of Horses	21	17	12	14	14	11	*p*-Value		
Physicochemical parameters									
*Musculus longissimus lumborum*									
pH_i_	6.49 ± 0.06 ^a^	6.43 ± 0.02 ^a^	6.94 ± 0.22 ^b^	6.48 ± 0.1 ^a^	6.29 ± 0.09 ^c^	6.69 ±0.10 ^d^	<0.0001	<0.0001	<0.0001
T_i_ (°C)	35.77 ± 2.28	36.02 ± 2.26	36.16 ± 2.52	36.19 ± 2.09	36.26 ± 2.36	34.91 ± 2.13	0.691	0.618	0.366
pH_u_	5.63 ± 0.12 ^a^	5.53 ±0.04 ^a^	6.00 ± 0.31 ^b^	5.53 ± 0.14 ^a^	5.59 ± 0.03 ^a^	5.81 ± 0.03 ^c^	0.021	<0.0001	0.005
T_u_ (°C)	3.40 ± 0.88	3.46 ± 0.98	3.70 ± 1.06	3.63 ±0.99	3.57 ± 1.04	2.96 ±0.32	0.509	0.717	0.134
*Musculus gracilis*									
pH_i_	6.54 ± 0.13	6.59 ± 0.22	6.51 ± 0.29	6.54 ± 0.14	6.49 ± 0.17	6.58 ± 0.18	0.800	0.959	0.278
T_i_ (°C)	38.40 ± 0.60	38.24 ± 0.96	38.02 ± 0.65	37.77 ± 0.35	37.34 ± 1.15	38.11 ± 0.74	0.004	0.050	0.306
pH_u_	5.67 ± 015	5.73 ± 0.14	5.71 ± 0.16	5.71 ± 0.09	5.72 ± 0.10	5.71 ± 0.06	0.867	0.490	0.747
T_u_ (°C)	5.89 ± 0.77	5.74 ± 1.07	5.80 ±0.83	5.99 ± 0.78	5.69 ± 0.96	5.76 ± 0.83	0.979	0.581	0.924
*Musculus longissimus lumborum*									
Water-holding capacity (%)									
Drip loss	1.77 ± 0.24 ^a^	1.80 ± 0.23 ^a^	0.56 ± 0.06 ^b^	2.39 ± 0.70 ^c^	2.80 ±1.05 ^c^	1.09 ± 0.02 ^d^	<0.0001	<0.0001	0.050
Thawing loss	6.97 ± 1.26 ^a^	5.58 ± 1.63 ^b^	3.95 ± 1.50 ^c^	7.19 ± 1.67 ^a^	6.81 ± 1.02	6.80 ± 1.22 ^a^	0.017	0.069	0.206
Cooking loss	23.61 ± 4.11	26.72 ± 5.28	23.84 ± 2.02	25.38 ± 3.07	25.46 ± 6.78	26.47 ± 5.43	0.308	0.404	0.268
Colour traits									
L* (lightness) value	32.79 ± 0.42 ^a^	35.72 ± 0.87 ^b^	27.04 ± 0.52 ^c^	34.32 ± 0.37 ^ab^	36.83 ± 0.42 ^b^	30.17 ± 0.11 ^d^	<0.0001	<0.0001	<0.0001
a* (redness) value	10.24 ± 3.04 ^a^	11.27 ± 0.32 ^ac^	14.44 ± 0.59 ^b^	10.23 ± 0.40 ^a^	12.06 ± 0.23 ^ac^	12.78 ± 0.21 ^ac^	0.383	<0.0001	0.017
b* (yellowness) value	7.13 ± 1.21 ^a^	7.81 ± 1.13 ^a^	3.87 ± 1.41 ^b^	7.30 ± 1.35 ^a^	8.47 ± 1.47 ^c^	5.42 ± 0.75 ^d^	0.004	<0.0001	0.132
Meat quality classes									
Acid meat (pH_u_ ˂ 5.4)	9.52	11.76	8.33	14.29	14.29	9.09	0.7430	0.5627	0.9928
Normal meat (5.4 ≤ pH ≤ 5.9)	85.72	88.24	58.33	85.71	85.71	81.82	0.7817	0.1935	0.3618
DFD-like meat (pH ≥ 6.0)	4.76 ^a^	0.00 ^a^	33.33 ^b^	0.00 ^a^	0.00 ^a^	9.09 ^a^	0.2247	0.0035	0.0045

Abbreviations: high loading density—space available in the transport vehicle higher over 200 kg/m^2^ (*n* = 50); low loading density—space available in the transport vehicle equal to or less than 200 kg/m^2^; pH_i_—meat pH values measured 45 min postmortem; T_i_—meat temperature measured 45 min postmortem; pH_u_—meat pH values measured 24 h postmortem; T_u_—meat temperature measured 24 h postmortem; drip loss—fluid loss at 4 °C for a period of 24 to 72 (Bag method); thawing loss—fluid loss during thawing at room temperature for 12–16 h; cooking loss—fluid loss during cooking until the internal temperature reached 75 °C; L* value—light reflectance; a* value—intensity of red/green colour; b* value—intensity of yellow/blue colour; DFD—dark, firm, and dry. Note: *p*-values correspond to two-way ANOVA test followed by Tukey’s multiple comparison test; different letters in the same row indicate a significant difference at *p* < 0.05 ^(a–d)^.

## Data Availability

The data that support the findings of this study are available from the corresponding author upon reasonable request.
